# Accelerated construction of stress relief music datasets using CNN and the Mel-scaled spectrogram

**DOI:** 10.1371/journal.pone.0300607

**Published:** 2024-05-24

**Authors:** Suvin Choi, Jong-Ik Park, Cheol-Ho Hong, Sang-Gue Park, Sang-Cheol Park

**Affiliations:** 1 College of General Education, Chung-Ang University, Seoul, Korea; 2 Department of Electrical and Computer Engineering, Carnegie Mellon University, Pittsburgh, PA, United States of America; 3 Department of Intelligent Semiconductor Engineering, Chung-Ang University, Seoul, Korea; 4 Department of Applied Statistics, Chung-Ang University, Seoul, Korea; 5 Artificial Intelligence and Robotics Laboratory, Myongji Hospital, Goyang, Korea; Nanjing Normal University, CHINA

## Abstract

Listening to music is a crucial tool for relieving stress and promoting relaxation. However, the limited options available for stress-relief music do not cater to individual preferences, compromising its effectiveness. Traditional methods of curating stress-relief music rely heavily on measuring biological responses, which is time-consuming, expensive, and requires specialized measurement devices. In this paper, a deep learning approach to solve this problem is introduced that explicitly uses convolutional neural networks and provides a more efficient and economical method for generating large datasets of stress-relief music. These datasets are composed of Mel-scaled spectrograms that include essential sound elements (such as frequency, amplitude, and waveform) that can be directly extracted from the music. The trained model demonstrated a test accuracy of 98.7%, and a clinical study indicated that the model-selected music was as effective as researcher-verified music in terms of stress-relieving capacity. This paper underlines the transformative potential of deep learning in addressing the challenge of limited music options for stress relief. More importantly, the proposed method has profound implications for music therapy because it enables a more personalized approach to stress-relief music selection, offering the potential for enhanced emotional well-being.

## 1 Introduction

Music listening is a mediation technique that is widely employed in clinical environments. Moreover, humans often listen to music in their daily lives to relieve stress, improve their mood, and conduct self-expression [1]. Among these purposes, relieving or managing stress has become crucial according to several studies that proved the effectiveness of music listening in such areas [2–4]. For example, Thoma et al. [2] examined the effects of listening to music on healthy women. The researchers played relaxing music to participants before a stressful task, and they exhibited different stress responses compared to the non-music control groups (p = 0.025). In addition, Linnemann et al. [3] researched 55 healthy university students, and the clinical trial results indicated that music listening effectively reduced subjective stress levels (p = 0.010). Other studies have continued to demonstrate that music is effective in managing stress. For example, a recent survey indicated that 42.7% of music therapists worldwide use music listening during therapeutic mediation [5].

Listening to music can evoke specific emotional states according to the content [6], and *stress-relief music* (SM) is qualified by several physical reactions during and after the music is played. Biological responses (such as blood pressure, skin temperature, and emotional changes) are measured during and after playing music to participants to confirm whether specific music can be classified as SM. From synthesizing the biological responses, the music is determined as SM if participants exhibit low arousal and high valence [7, 8]. Moreover, participants can leverage improved SM benefits if their regional and cultural characteristics and preferences are considered [9, 10]. However, selecting SM from existing music is both time- and cost-consuming due to the requirement of experimental verification. Moreover, selecting SM that reflects the subjects’ stances makes the adequate SM insufficient.

Rahman et al. [8] innovated in SM selection by using deep learning to process biological responses, achieving over 95% test accuracy with *convolutional neural networks* (CNNs). However, their approach was limited by the need for specialized equipment to measure these responses, leading to time and cost constraints. On a different front, Abboud et al. [11, 12] extracted features directly from music using fuzzy k-nearest neighbors (KNN), but faced scalability issues with large, high-dimensional datasets. These limitations highlight the advantages of CNN models, which don’t require extensive data storage for making inferences and are more efficient for classifying SM.

The core objective of our study is to examine the practicality of constructing SM datasets utilizing CNNs without reliance on biological response measurements. We propose leveraging the *elements of music* (EM)—such as pitch, rhythm, melody, timbre, and dynamics—as indicators of a song’s potential for stress relief. These EMs are harmonized expressions of the underlying *elements of sound* (ES), which include frequency, amplitude, and waveform.

Historically, research has connected biological responses with the emotional states evoked by music, specifically in terms of valence and arousal, as indicated in studies by Russell et al. [7] and Rahman et al. [8]. Further, Droit-Volet et al. [13] identified emotional states through the analysis of EMs, particularly tempo. Abboud et al. [11, 12] observed that classification performance improves when models are trained on a larger number of music features directly extracted from the audio. We posit an inductive relationship between ES and the emotional states evoked by music, which can be represented as follows:

$$\begin{array}{c} \text{Evoked}\;\text{Emotional}\;\text{States}=f(EM)=f(g(ES))=F(ES), \end{array}$$

where *g* is the transformation function that maps ES to EM, *f* represents the function that correlates EMs with emotional states, and *F* is the composition of these functions, directly relating ES to emotional states. Although deriving an explicit formula for this relationship is challenging, we can approximate it using CNNs, which are recognized for their ability to model complex, non-linear relationships in data [14–16].

In our CNN model, we strategically choose to employ the *Mel-scaled spectrogram* (MSS) [17] as a pivotal feature. This decision is bolstered by the MSS’s proven superior performance in music genre classification tasks when used in conjunction with CNNs, highlighting its potential effectiveness for our purposes [18]. The Mel scale is specifically designed to mirror human auditory sensitivity, adeptly capturing variations in frequency and amplitude within audio signals [19]. This congruence with the nuances of human hearing renders the MSS an exceptionally effective tool for dissecting the emotional impacts embedded in music, a core aspect of identifying SM. Moreover, the MSS’s ability to transform sound frequencies into a perceptually relevant scale offers a nuanced and detailed musical representation. This feature is vital for our CNN model, as it enables a more precise interpretation of the emotional nuances conveyed by various musical elements. Notably, the MSS has the frequency and amplitude information (i.e., ES) thereby providing a comprehensive auditory profile essential for our analysis.

This paper provides two main contributions:

- To the best of our knowledge, this is the first deep learning approach using ES to improve time efficiency and reduce costs compared to measuring biological responses when constructing SM datasets.
- The trained CNN model, which includes a classifier for distinguishing SM, can sort data of unlabeled music of various genres (such as hip-hop, rock, classical, and blues). Through this approach, large-scale SM datasets can sufficiently reflect participants’ regional and cultural characteristics and preferences and increase the effectiveness of music listening [9, 10].

In this paper, we discuss previous related music classification studies in Section 2, our SM classification method is described in Section 3, the experimental evaluation (with the clinical study) is presented in Section 4, and the conclusions are provided in Section 6.

## 2 Related works

### 2.1 Music emotion recognition

**Fig 1:**
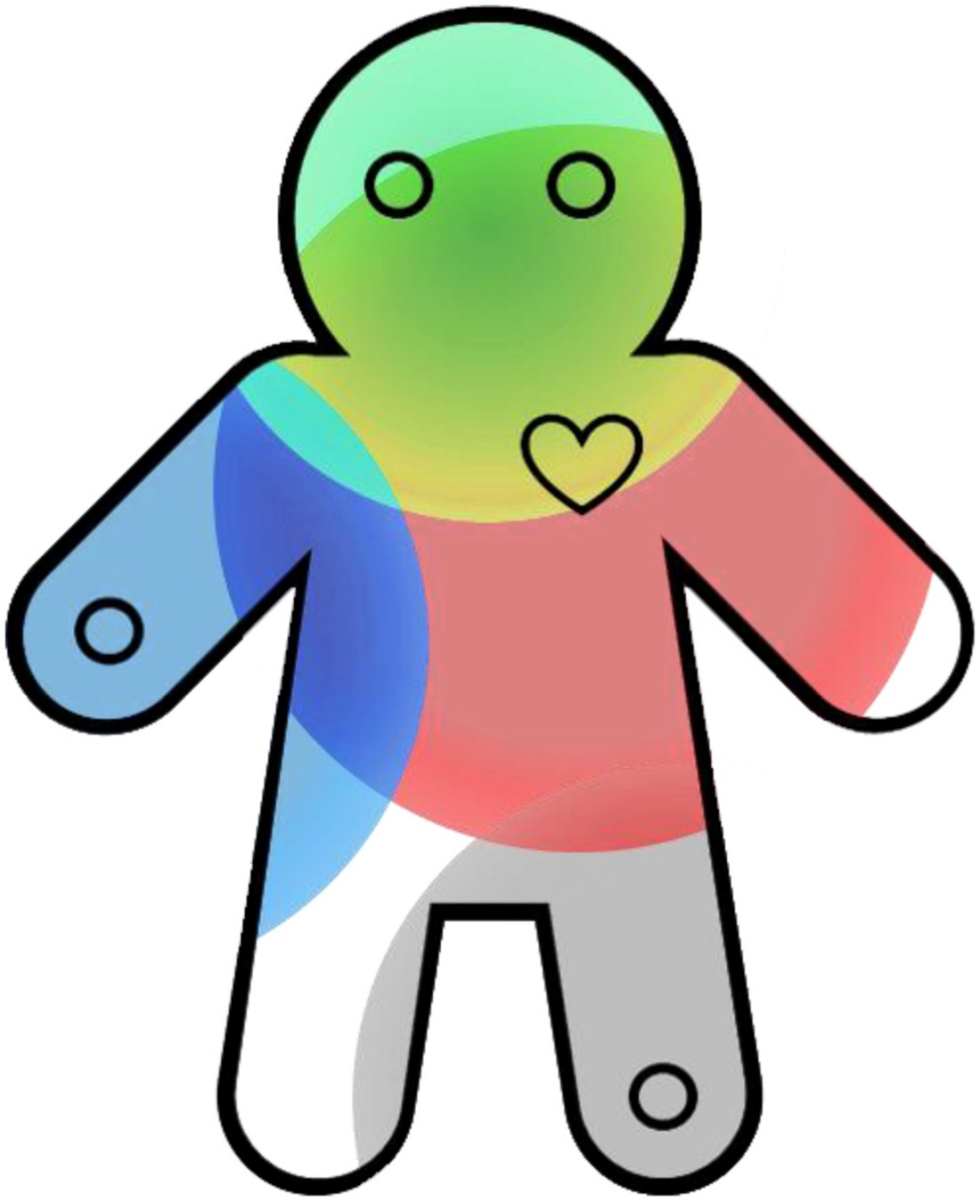
Human-shaped image indicating pupil dilation, electrodermal activity, blood volume pulse, and skin temperature of participants. To explain the study [8], this image was created by only mimicking the shape of the original image, and it differs from the image actually used for training. The original image can be accessed under the Creative Commons Attribution-NonCommercial-NoDerivatives 4.0 International license.

To classify emotions evoked from music listening, Rahman et al. [8] initially measured the pupil dilation, electrodermal activity, blood volume pulse, and skin temperature of participants. Then, these features were visualized by placing them at four vertices on human-shaped blank images, as depicted in Fig 1.

In the figure, the features are represented by rings with different colors, and the ring sizes vary according to the degree of each influence. The images were labeled according to their evoked emotional state and then used to train the CNN models. The test results of the CNN models were higher than other machine learning techniques (such as KNN and support vector machine). Even though Rahman et al. demonstrated that CNN approaches are superior to other methods for classifying emotions evoked from music listening, there were cost and time limitations because the classification model could only be used after the body responses of the participants had been measured. Our study solves the limitations of this previous study [8] by using a classification method that only uses ES (such as frequency, amplitude, and waveform) obtained from music. Instead of body responses, we used the MSSs presented in Section 2.2 (which were directly converted from a song), and we utilized the original information of the music (i.e., ES).

Abboud et al. [11, 12] conducted a study in which features were directly extracted from music. The method they used was fuzzy KNN, which is a machine-learning algorithm. However, there was a limitation in that the computational performance of Fuzzy KNN can degrade with large datasets (particularly high-dimensional data) because it requires the storing of all training data for predictions. This issue poses challenges for making objective inferences with large amounts of data, which are often needed to improve the accuracy of stress management music classification. As the authors noted, a decrease in mean squared error was observed when increasing the size of the data, indicating a potential improvement in performance with larger datasets. However, despite these efforts, the need to handle large and high-dimensional datasets highlights the potential limitations of the fuzzy KNN approach. Therefore, this paper suggests using CNNs (a deep learning approach), which can provide an alternative solution due to the ability to effectively manage and learn from large, high-dimensional datasets. This is possible because the CNN approach only requires a trained model to make inferences [14, 20, 21].

### 2.2 Music genre classification with Mel-scaled spectrogram

**Fig 2:**
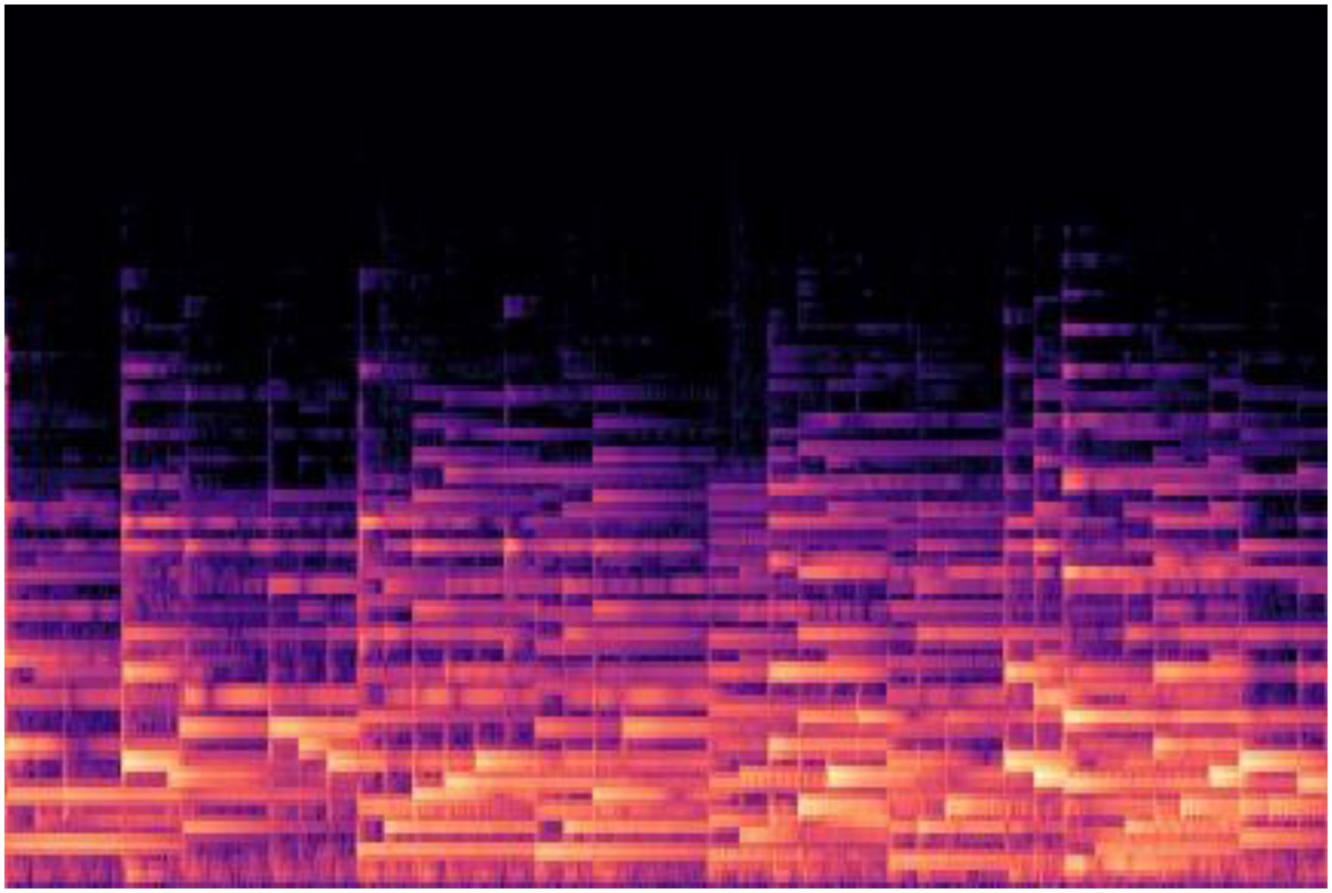
A Mel-scaled spectrogram generated from a song. On the x-axis, we have the time dimension, representing the duration of the audio segment. The y-axis denotes the frequency. The color intensity in the spectrogram indicates the amplitude (or energy) of different frequencies at each point in time, with warmer colors representing higher amplitudes and cooler colors indicating lower amplitudes.

In terms of classifying music genres, one study trained CNN models with the Mel-scaled spectrogram (MSS) as a dataset, which exhibited superior performance compared to other machine-learning techniques with different data formats in previous studies [18]. The MSS is a type of spectrogram with the Mel scale on the y-axis [17]. The Mel scale was designed to make detecting sound information at lower frequencies easier than at higher frequencies. The MSS representation captures essential acoustic features relevant to genre classification, and the Mel scale emphasizes the lower frequencies, which often carry key distinguishing features for different music genres. This characteristic becomes crucial for SM classification as these genres often have unique ES compositions. As depicted in Fig 2, an MSS expresses ES for a specific period as an image. Droit-Volet et al. [13] categorized evoked emotional states by analyzing tempo, implying that ES infers emotional states and MSS represents emotions. Our study indicated that SM classification tasks can leverage the capabilities of MSS, suggesting that the CNN model can serve as a cost-effective and efficient classifier for SM.

### 2.3 DEAM for cross-validation of the CNN model

The Database for Emotional Analysis of Music (DEAM) consists of 1,802 songs and is a representative music dataset that analyzes arousal and valence in seconds through an experimental method [22]. DEAM provides valence and arousal values for each song, which are recorded every second while they are being played. In our study, we first calculated the average valence and arousal values for each song across its entire duration to obtain a consistent metric for comparison. Based on the established understanding that music conducive to stress relief typically exhibits low arousal and high valence, we categorized songs within the DEAM dataset accordingly. Specifically, songs demonstrating an negative average arousal and an positive average valence were classified as SM, amounting to 212 songs. Conversely, songs not meeting these criteria, totaling 1,590, were categorized as non-SM.

Herein, we propose a method for constructing SM datasets quickly and cost-effectively, highlighting that providing SM that reflects participants’ regional and cultural characteristics is more effective in relieving stress [9, 10]. However, the amount of SM reflecting such properties was insufficient. Therefore, we trained the CNN model with songs that effectively relieved stress in Koreans according to previous studies (i.e., a custom dataset) [4, 23–26]. Accordingly, since this custom dataset might not meet the general standard of SM, we classified DEAM using the CNN model trained with the custom dataset and confirmed that the CNN model is objective by checking the classification accuracy.

## 3 Design

### 3.1 Training CNN model

**Fig 3:**
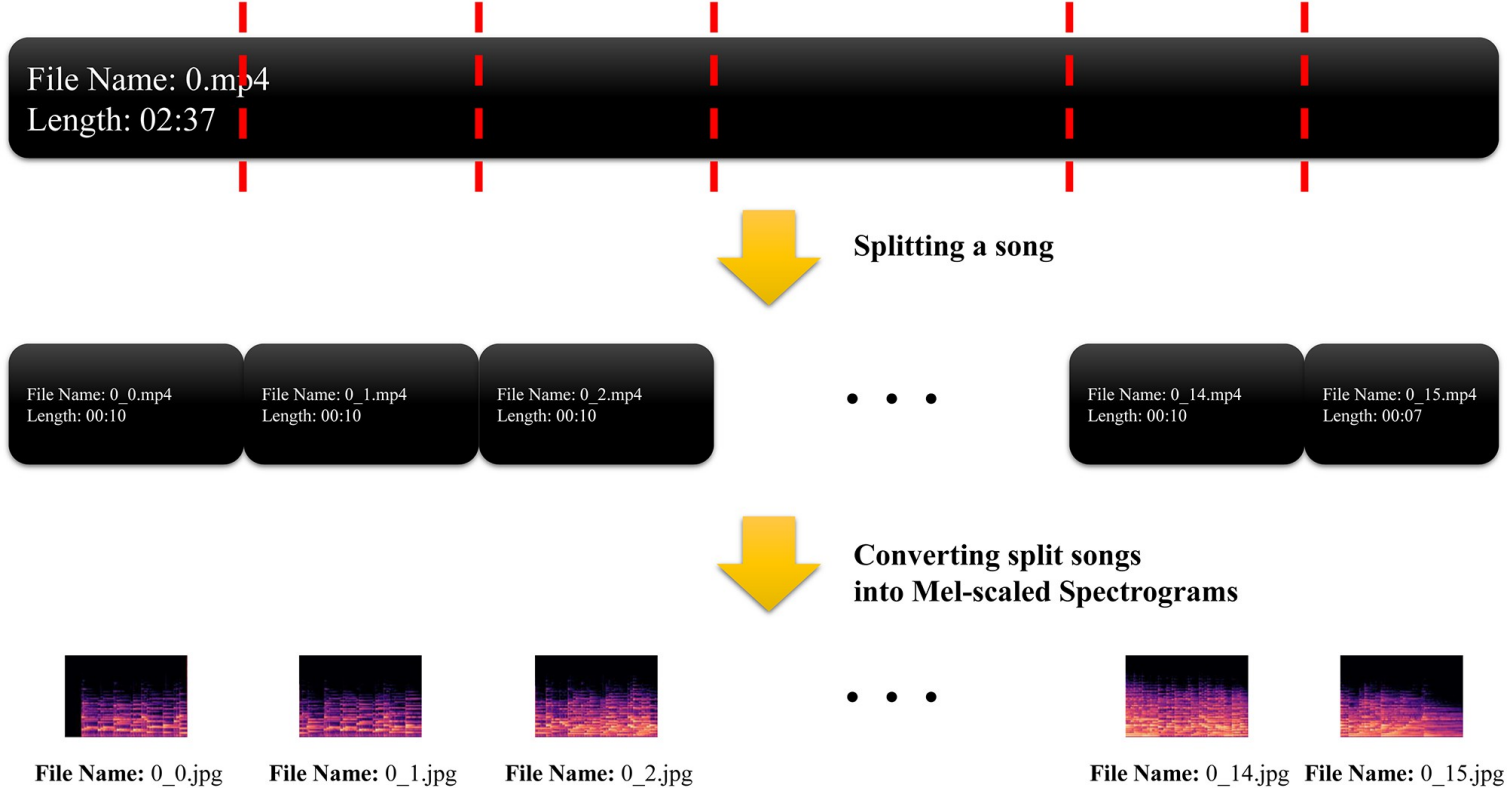
The process of converting a song into Mel-scaled spectrograms. Initially, the song is segmented into discrete units, each spanning 10 seconds. Subsequently, each of these 10-second segments is individually transformed into a Mel-scaled spectrogram.

To train the CNN model, we utilize a custom dataset comprising 50 songs from previous studies [4, 23] that were determined as effective in relieving stress in Koreans (i.e., SM) and 58 songs that were non-SM. The rationale behind this selection is to ensure that our model is trained on a balanced dataset that accurately reflects a variety of musical attributes associated with both stress relief and non-stress relief categories. This balanced approach helps to avoid bias in the model’s predictions, considering that an unbalanced dataset could skew the model’s learning, leading to overfitting to the characteristics of the predominant class [27]. These 108 songs are divided into 10 s units of 44,100 Hz and then converted into 2,901 MSSs, comprising 1,366 for SM and 1,535 for non-SM. Fig 2 presents a sample of a converted MSS, and the transformation of a song into MSSs is depicted in Fig 3. To convert each unit of the songs into MSSs, the process begins with the extraction of short-term Fourier transform (STFT) from the audio signal [17]. STFT decomposes the signal into its frequency components, providing a time-frequency representation. This transformation is critical for capturing the temporal dynamics of the music. The mathematical formula for STFT is given by:

$$\begin{array}{c} STFT\left(t,\omega \right)=\int x\left(\tau \right)w\left(\tau -t\right)e^{-j\omega \tau }d\tau \end{array}$$

where *x*(*τ*) is the signal, *w*(*τ* − *t*) is the window function centered around time *t*, and *ω* is the frequency. Following STFT, the frequency bins are then mapped onto the Mel scale, a perceptual scale of pitches judged by listeners to be equal in distance from one another. This mapping is achieved through a Mel filter bank, which converts the frequency scale into the Mel scale, effectively capturing the human ear’s non-linear perception of sound. The Mel frequency is calculated using the formula:

$$\begin{array}{c} m=2595\;{\text{log}}_{10}\left(1+\frac{f}{700}\right) \end{array}$$

where *m* is the Mel frequency and *f* is the linear frequency.

For convenience, we used the *librosa* [28] library which automates this process in Python.

DEAM is also transformed into MSSs and consisted of 1,802 songs. Among these songs, 212 with low arousal and high valence are labeled SM, and the remaining 1,590 are labeled non-SM. Since approximately 95% of the songs in DEAM last 45 s, MSSs are only converted up to 45 s for songs exceeding this length. Table 1 depicts the custom and the DEAM datasets’ sample sizes for this study.

**Table 1:** Dataset information includes the number of songs and the number of Mel-scaled spectrograms converted from songs. SM and Non-SM stand for stress relief music and non-stress relief music, respectively.

		# of songs	# of Mel-scaled Spectrograms
**Custom Dataset**	**SM**	50	1,366
	**Non-SM**	58	1,535
**DEAM Dataset**	**SM**	212	1,060
	**Non-SM**	1,590	7,950

All MSSs have a height of 288 pixels and a width of 432 pixels, which qualifies them as large-scale images. For the classification of such images, various CNN architectures have been introduced, with Residual Networks (ResNets) and Dense Convolutional Networks (DenseNets) being prominent examples. He et al. [15] introduced ResNets, where architectures like ResNet-18, ResNet-50, and ResNet-101 have shown effectiveness in deep learning tasks. ResNet models are characterized by their depth (18, 50, and 101 layers, respectively) and the use of residual blocks that facilitate the training of these deep networks by allowing the bypassing of certain layers. Similarly, DenseNets [29], particularly DenseNet-161, 169, and 201, have also gained attention due to their unique approach of connecting each layer to every other layer in a feed-forward fashion. This design ensures maximum information flow between layers, enhancing feature propagation and reducing the number of parameters. Table 2 provides detailed structures of ResNet-18, 50, 101, and DenseNet-161, 169, 201, respectively. These tables illustrate the layer configurations, kernel sizes, and channel dimensions for each network. In our study, we explored the use of both ResNet and DenseNet architectures for classifying SM images derived from MSSs.

**Table 2:** Structures of a) ResNet-18, 50, 101 and b) DenseNet-161, 169, 201. The architectural structures of two types of convolutional neural network models: a) ResNet and b) DenseNet. Specifically, it details the layer configurations, kernel sizes, and channel dimensions for three variants of ResNet (ResNet-18, ResNet-50, ResNet-101) and three variants of DenseNet (DenseNet-161, DenseNet-169, DenseNet-201).

**a)**			
**Layer Name**	**ResNet-18**	**ResNet-50**	**ResNet-101**
Convolution	7 × 7, 64, stride 2		
Pooling	3 × 3 max pool (stride 2)		
Residual Block (1)	$[\begin{array}{c} 3\times 3,64\\ 3\times 3,64 \end{array}]\times 2$	$[\begin{array}{c} 1\times 1,64\\ 3\times 3,64\\ 1\times 1,256 \end{array}]\times 3$	$[\begin{array}{c} 1\times 1,64\\ 3\times 3,64\\ 1\times 1,256 \end{array}]\times 3$
Residual Block (2)	$[\begin{array}{c} 3\times 3,128\\ 3\times 3,128 \end{array}]\times 2$	$[\begin{array}{c} 1\times 1,128\\ 3\times 3,128\\ 1\times 1,512 \end{array}]\times 4$	$[\begin{array}{c} 1\times 1,128\\ 3\times 3,128\\ 1\times 1,512 \end{array}]\times 4$
Residual Block (3)	$[\begin{array}{c} 3\times 3,256\\ 3\times 3,256 \end{array}]\times 2$	$[\begin{array}{c} 1\times 1,256\\ 3\times 3,256\\ 1\times 1,1024 \end{array}]\times 6$	$[\begin{array}{c} 1\times 1,256\\ 3\times 3,256\\ 1\times 1,1024 \end{array}]\times 23$
Residual Block (4)	$[\begin{array}{c} 3\times 3,512\\ 3\times 3,512 \end{array}]\times 2$	$[\begin{array}{c} 1\times 1,512\\ 3\times 3,512\\ 1\times 1,2048 \end{array}]\times 3$	$[\begin{array}{c} 1\times 1,512\\ 3\times 3,512\\ 1\times 1,2048 \end{array}]\times 3$
Classification Layer	average pool, 2-d fully connected, softmax		
**b)**			
**Layer Name**	**DenseNet-121**	**DenseNet-169**	**DenseNet-201**
Convolution	7 × 7, 64, stride 2		
Pooling	3 × 3 max pool (stride 2)		
Dense Block (1)	$[\begin{array}{c} 1\times 1,128\\ 3\times 3,32 \end{array}]\times 6$	$[\begin{array}{c} 1\times 1,128\\ 3\times 3,32 \end{array}]\times 6$	$[\begin{array}{c} 1\times 1,128\\ 3\times 3,32 \end{array}]\times 6$
Transition Layer (1)	1 × 1 convolutional layer, average pool (stride 2)		
Dense Block (2)	$[\begin{array}{c} 1\times 1,128\\ 3\times 3,32 \end{array}]\times 12$	$[\begin{array}{c} 1\times 1,128\\ 3\times 3,32 \end{array}]\times 12$	$[\begin{array}{c} 1\times 1,128\\ 3\times 3,32 \end{array}]\times 12$
Transition Layer (2)	1 × 1 convolutional layer, average pool (stride 2)		
Dense Block (3)	$[\begin{array}{c} 1\times 1,128\\ 3\times 3,32 \end{array}]\times 24$	$[\begin{array}{c} 1\times 1,128\\ 3\times 3,32 \end{array}]\times 32$	$[\begin{array}{c} 1\times 1,128\\ 3\times 3,32 \end{array}]\times 48$
Transition Layer (2)	1 × 1 convolutional layer, average pool (stride 2)		
Dense Layer (4)	$[\begin{array}{c} 1\times 1,128\\ 3\times 3,32 \end{array}]\times 16$	$[\begin{array}{c} 1\times 1,128\\ 3\times 3,32 \end{array}]\times 32$	$[\begin{array}{c} 1\times 1,128\\ 3\times 3,32 \end{array}]\times 32$
Classification Layer	average pool, 2-d fully connected, softmax		

After training the CNN models, we classify the MSSs of DEAM to verify that the CNN models are trained objectively.

### 3.2 Clinical study

By employing the verified CNN model with DEAM, we filter the top 10 most popular Korean songs from each of 12 distinct genres, amounting to a total of 220 songs. However, considering the overlaps in song selections across these genres, the final count stands at 164 unique songs. Detailed lists of these songs can be found in Tables 8–10 in the S1 Appendix.

We then select 5 songs that exhibit the highest SM matching rate for the clinical study to confirm that the CNN model is applicable in real-world situations. The SM matching rate is calculated using Eq 1 because each song had multiple MSSs.

$$\begin{array}{c} Matching\;Rate=\frac{The\;Number\;of\;Matched\;MSSs}{The\;Number\;of\;All\;MSSs}. \end{array}$$ (1)

In Eq 1, the number of matched MSSs refers to the number of classified MSSs as an SM of a song, and the number of all MSSs refers to the number of all converted MSSs of a song.

In the clinical study, comparing non-SM and SM would not obtain accurate experimental results because studies have claimed that individual favorite music (IM) helps to relieve and manage stress [30–32], and non-SM could include IM. Moreover, the clinical study demonstrates that researcher-selected music (RM) with the CNN model was not inferior to the stress-relieving effects of IM.

**Fig 4:**
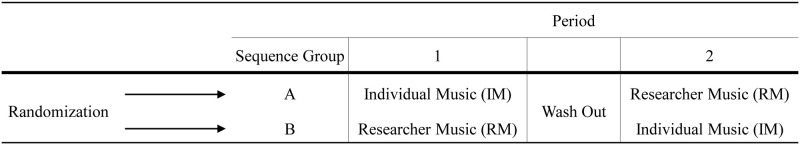
The design of the clinical study employing a 2 × 2 crossover methodology. Participants were randomized into two sequence groups, A and B. Group A first experienced Individual Music (IM) followed by Researcher-selected Music (RM) after a washout period. Conversely, Group B started with RM and then transitioned to IM, also separated by a washout period.

The clinical study was a 2 × 2 crossover design consisting of random, 2-sequence, 2-period, and 2-treatment, as shown in Fig 4. The participants in the clinical study were randomly assigned to the A sequence (IM-RM) and B sequence groups (RM-IM). The clinical study contained a 40-min washout period for the participants placed between Periods 1 and 2, considering that the treatment of Period 1 would affect the treatment of Period 2. We confirmed that there was no residual effect between treatments. Before Period 1, after Period 1, and after Period 2, the participants responded with discrete visual analog scale (VAS) scores for three emotional states: stress, happiness, and satisfaction. The clinical study utilized VAS values ranging from 0 to 10 to evaluate these states. Herein, VAS is a line composed of 10 cm long horizontal lines. It should be noted that VAS can minimize the researcher’s involvement and is used extensively in clinical environments because it allows participants to express their subjective emotions and pain [33, 34]. The hypotheses for the three emotional state responses were obtained through the 2 × 2 crossover design experiment and are represented in Eq 2 where *μ*_*RM*_ and *μ*_*IM*_ represent the averages in the population of the listening RM and IM groups, respectively. The null hypothesis was tested to determine whether the lower boundary of the 95% confidence interval exceeded 80%.

$$\begin{array}{c} \begin{array}{ccc} & H_{0}:\frac{\mu_{RM}}{\mu_{IM}}\leq log\left(0.8\right)\;\; & H_{1}:\frac{\mu_{RM}}{\mu_{IM}}>log\left(0.8\right). \end{array} \end{array}$$ (2)

Of the 90 volunteers for this study, 80 fulfilled the selection criteria. These criteria excluded people with hearing loss problems and any who had taken drugs for neurological/psychiatric diseases or chronic pain within the last year because participants had to respond to the emotional states (stress, happiness, and satisfaction) after music listening. The clinical study was conducted after being reviewed and approved by the Research Ethics Review Committee (IRB No.1041078-201907-HR-217-01) at Chung-Ang University. The purpose of the clinical study, research procedure, and compensation details were explained to the participants, who fully understood the risks and benefits of participating. We also explained in detail and guaranteed that all personal information would not be used for any purposes other than this research. Table 3 displays the participants’ basic biological information (age and sex). All the participants verbally agreed to participate in the clinical study, and there were no minors involved.

**Table 3:** A summary table of the participants’ basic biological information, categorized by age and sex. It displays the mean and median ages, the age range (minimum and maximum values), and the distribution of participants by sex for each sequence group of the clinical study.

		1. A → B (*N* = 39)	2. B → A (*N* = 41)	p-value
**Age**	Mean (SD)	37.8 (7.91)	38.2 (8.49)	0.818
	Median [Min, Max]	40.0 [25.0, 50.0]	36.0 [25.0, 54.0]	
**Sex**	Male	17 (43.6%)	20 (48.8%)	0.809
	Female	22 (56.4%)	21 (51.2%)	

The participants responded to their emotional states before the treatment. Table 4 presents these baseline demographics to confirm the degree of change in emotional states after listening to RM and IM.

**Table 4:** A table of baseline demographics, detailing the initial levels of stress, happiness, and satisfaction among participants before the clinical study commenced. It includes mean and median values, as well as the range (minimum and maximum scores) for each emotional state across the two sequence groups.

		1. A → B (*N* = 39)	2. B → A (*N* = 41)	p-value
**Stress**	Mean (SD)	6.21 (1.26)	6.10 (1.84)	0.760
	Median [Min, Max]	6.00 [3.00, 9.00]	7.00 [3.00, 10.00]	
**Happiness**	Mean (SD)	5.38 (1.65)	5.51 (1.58)	0.725
	Median [Min, Max]	5.00 [2.00, 9.00]	5.00 [3.00, 9.00]	
**Satisfaction**	Mean (SD)	4.95 (1.61)	4.90 (1.87)	0.906
	Median [Min, Max]	5.00 [2.00, 8.00]	5.00 [1.00, 8.00]	

## 4 Model and clinical study evaluation

### 4.1 CNN model training

The training was conducted on 4 GPUs of a DGX-V100. We evaluated six different network architectures: ResNet-18, ResNet-50, ResNet-101, DenseNet-161, DenseNet-169, and DenseNet-201. Each model was trained with a mini-batch size of 8, using a stochastic gradient descent optimizer with an initial learning rate of 0.1 and momentum of 0.9. A cosine annealing scheduler was employed to reduce the learning rate from 0.1 to 0.001 over 200 epochs. During training and inference, MSSs, converted from songs, served as input data. Data augmentation techniques, other than normalization (mean and standard deviation set to 0.5), were not applied to the MSSs, as each part of the MSS contained essential ES.

**Fig 5:**
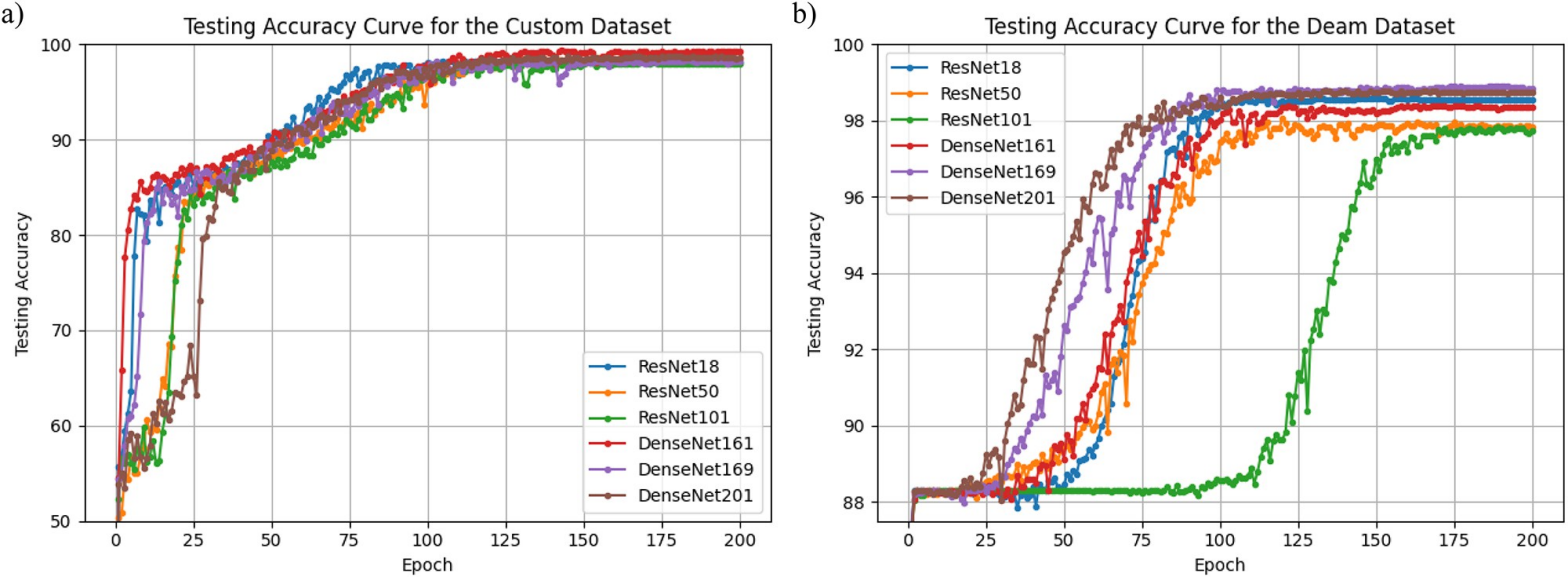
The comparative testing accuracy curves for ResNet-18, ResNet-50, ResNet-101, DenseNet-161, DenseNet-169, and DenseNet-201 models, using both custom and DEAM datasets. The curves illustrate how the accuracy rates of each model vary over the testing period.

After training, all CNN models achieved test accuracies above 98.1% for the custom dataset. The testing accuracies across 200 epochs for the custom dataset are depicted in Fig 5a) and summarized in Table 5.

**Table 5:** A comprehensive summary of testing accuracy, F1-score, Recall, and Precision metrics for the custom and DEAM datasets, as evaluated across a range of models including ResNet-18, ResNet-50, ResNet-101, DenseNet-161, DenseNet-169, and DenseNet-201.

**Custom Dataset**							
**Model**	**Accuracy**	**F1-score**		**Recall**		**Precision**	
		**SM**	**Non-SM**	**SM**	**Non-SM**	**SM**	**Non-SM**
ResNet-18	98.7%	0.916	0.919	0.891	0.944	0.942	0.894
ResNet-50	98.6%	0.932	0.930	0.923	0.940	0.942	0.920
ResNet-101	98.1%	0.912	0.898	0.890	0.924	0.934	0.874
DenseNet-161	99.4%	0.971	0.967	0.956	0.985	0.987	0.949
DenseNet-169	98.4%	0.922	0.916	0.918	0.921	0.927	0.911
DenseNet-201	98.8%	0.940	0.936	0.924	0.953	0.956	0.920
**DEAM Dataset**							
**Model**	**Accuracy**	**F1-score**		**Recall**		**Precision**	
		**SM**	**Non-SM**	**SM**	**Non-SM**	**SM**	**Non-SM**
ResNet-18	98.6%	0.960	0.648	0.985	0.529	0.937	0.837
ResNet-50	98.1%	0.947	0.407	0.986	0.283	0.912	0.723
ResNet-101	97.8%	0.941	0.373	0.974	0.274	0.910	0.586
DenseNet-161	98.4%	0.956	0.584	0.980	0.472	0.932	0.765
DenseNet-169	98.9%	0.970	0.711	0.984	0.622	0.955	0.830
DenseNet-201	98.8%	0.966	0.700	0.990	0.577	0.944	0.888

Among the CNN architectures tested, we opted for ResNet-18 as our model of choice due to its efficiency and relatively lightweight architecture. Notably, ResNet-18’s testing accuracy was found to be comparable to the other models, deviating by less than 2% from the results obtained with the custom dataset.

Additionally, to address potential biases of the custom dataset and validate our model’s objectivity, we applied the trained ResNet-18 model to the DEAM dataset [22], a widely used resource for emotional analysis in music [35, 36]. The classification accuracy achieved on the DEAM dataset was 80.0%.

The ResNet-18 model, trained with the custom dataset, was subsequently employed to classify 164 unique songs. These songs were chosen as the top 10 most popular Korean songs from each of 12 distinct genres, ensuring there was no overlap in the selection. Using the matching rate formula (Eq 1), we identified songs with a matching rate exceeding certain thresholds. Specifically, 41 songs had a matching rate over 0.5, 9 songs had a matching rate over 0.9, and only 6 songs achieved a matching rate over 0.95. Based on these classification results, we selected the top 5 songs with the highest matching rates for use in our clinical study. These findings also suggest that identifying suitable SM across all genres is a challenging task, as appropriate SM constituted about 10% of the total music analyzed. This indicates that SM likely possesses unique characteristics that set it apart from other music.

### 4.2 Clinical study

The 80 participants listened to 1 song from the 5 songs with the highest SM matching rate (i.e., RM), and 1 song from the 5 individual favorite songs (i.e., IM) in Periods 1 and 2 of Fig 4. The order of listening to the two songs varied depending on whether it was Group A or B. The participants had a 40-min washout period between Periods 1 and 2 to prevent the treatment of Period 1 from affecting the treatment of Period 2.

**Fig 6:**
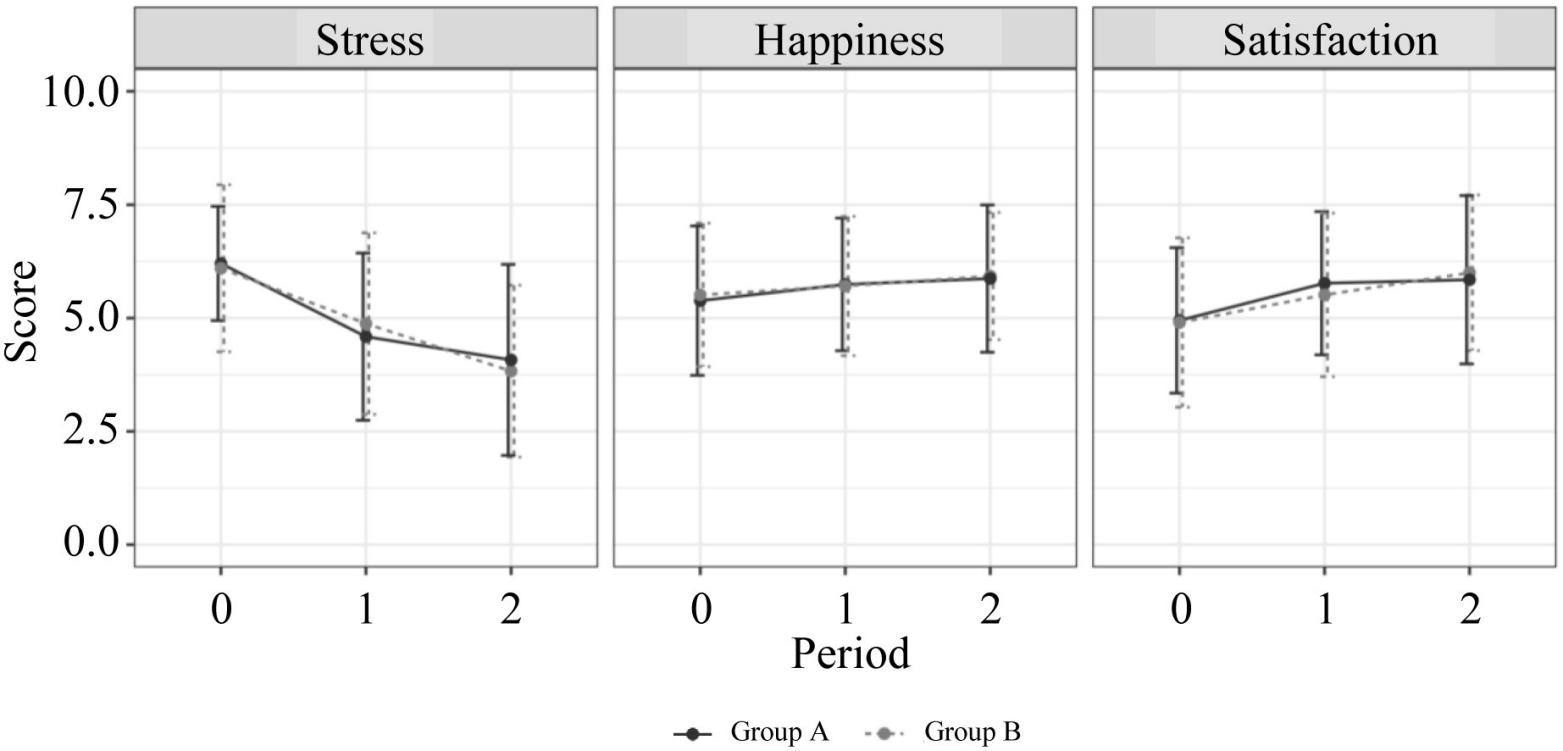
The distribution of Visual Analog Scale (VAS) scores for stress, happiness, and satisfaction, measured before and after the clinical test. It provides a visual comparison of the emotional state changes experienced by participants as a result of the intervention.

Fig 6 displays the VAS scores of stress, happiness, and satisfaction at the baseline, Period 1, and Period 2. Period 0 was the baseline, and the therapeutic effects decreased as the periods progressed (0→1→2). The VAS scores for stress decreased by 2.13 and 2.27, the VAS scores for happiness increased by 0.47 and 0.42, and the VAS scores for satisfaction increased by 0.9 and 1.1 in Groups A and B, respectively.

The increases or decreases in VAS scores according to emotional states are displayed in Table 6. Each feature was denoted with the *mean (standard deviation)* of all participants’ VAS scores, and features of pre- and post-columns depict changes in VAS scores before and after treatments.

**Table 6:** The VAS scores for stress, happiness, and satisfaction before and after the clinical test. The data is summarized to show the mean and standard deviation of participants’ scores, highlighting the changes in emotional states prompted by the clinical intervention.

		RM		IM	
		Pre	Post	Pre	Post
**Stress**	**Group 1**	6.21 (1.26)	4.59 (1.85)	4.59 (1.85)	4.08 (2.11)
	**Group 2**	4.88 (2.00)	3.83 (1.90)	6.10 (1.84)	4.88 (2.00)
	**Total**	5.53 (1.80)	4.20 (1.90)	5.63 (1.98)	4.48 (2.08)
**Happiness**	**Group 1**	5.38 (1.65)	5.74 (1.46)	5.74 (1.46)	5.85 (1.86)
	**Group 2**	5.71 (1.54)	5.93 (1.40)	5.51 (1.58)	5.71 (1.54)
	**Total**	5.55 (1.59)	5.84 (1.43)	5.63 (1.52)	5.79 (1.57)
**Satisfaction**	**Group 1**	4.95 (1.61)	5.77 (1.58)	5.77 (1.58)	5.85 (1.86)
	**Group 2**	5.51 (1.80)	6.00 (1.72)	4.90 (1.87)	5.51 (1.80)
	**Total**	5.24 (1.72)	5.89 (1.65)	5.33 (1.78)	5.68 (1.83)

Table 7 displays the non-inferiority test results. The upper and lower limits of the variables were [− 0.2180, 0.3901] for stress, [−0.0727, 0.0385] for happiness, and [−0.1232, −0.0050] for satisfaction, with p-values of 0.5253, 0.5418, and 0.0704, respectively. Therefore, we confirmed the null hypothesis, and RM was not inferior to IM.

**Table 7:** The results of the non-inferiority test, comparing the effectiveness of Researcher Music (RM) to Individual Music (IM) based on stress, happiness, and satisfaction scores. The data includes estimated means, differences between means, confidence intervals, p-values, and the assessment of non-inferiority.

	RM Estimated Mean (%)	IM Estimated Mean (%)	Difference Between Means [95% CI Limits]	p-value	Non-inferiority
**Stress**	1.1073	1.1934	0.0861	0.5253	True
			[-0.2180, 0.3901]		
**Happiness**	1.7359	1.7188	-0.0171	0.5418	True
			[-0.0727, 0.0385]		
**Satisfaction**	1.7296	1.6705	-0.0591	0.0704	True
			[-0.1232, -0.0050]		

## 5 Discussion

### 5.1 Validation of the training method

We also confirmed whether training the CNN models with MSSs for SM classification tasks were valid. After converting the 1,802 songs in DEAM to MSSs, we trained the same CNN models with the custom-dataset-trained CNN models. Here, we used DEAM’s MSSs, and the test accuracy were from 94.0%-to 98.9% (94.0% representing the worst case for ResNet-18 by varying hyper-parameters), which suggested that training the CNN models with MSSs was valid because the ratio of SM and non-SM was 1.5: 8.5 [37]. The testing accuracies for 200 epochs of the DEAM dataset are displayed in Fig 5(b).

### 5.2 Validation of the CNN model

As discussed in Section 4, the models trained on our custom dataset achieved upto a test accuracy of 99.4%. This high accuracy is partly due to the dataset’s robustness, which included music with proven stress-relief effects from prior clinical studies [4, 23]. However, the small size of the custom dataset might have contributed to this high accuracy due to limited data diversity. When applied to the larger and more varied DEAM dataset, the model’s accuracy decreased to 80.0%.

The DEAM dataset’s lower test accuracies for the DEAM-trained models compared to the custom-data trained models are primarily attributed to its binary labeling method for arousal and valence, based on averages above or below zero. This approach, which can be imprecise for values near zero, likely impacted the model’s accuracy. In comparison, the custom dataset’s more precise labeling criteria enabled better learning and generalization of SM patterns by the CNN model.

To mitigate the risk of overfitting, given the high accuracy with the custom dataset, we explored various CNN architectures and hyper-parameters. These included ResNet-50, ResNet-101, DenseNet-121, DenseNet-169, and DenseNet-201, along with learning rates ranging from 0.1 to 0.001 and momentum values of 0.5 and 0.9. The test accuracies varied by less than 2% from the results in Section 4. Training with the DEAM dataset showed that most models achieved over 98.0% testing accuracy as depicted in Fig 5b) and Table 5, confirming the robustness of the training methods. In this study, we selected ResNet-18 due to its lightweight architecture, making it broadly applicable regardless of computational constraints.

Despite the DEAM dataset’s labeling imprecisions and its larger size, the high accuracy achieved on the custom dataset validates the effectiveness of our CNN models. Furthermore, the balanced 1:1 ratio of SM to non-SM in the custom dataset underscores the model’s robustness.

### 5.3 Stress, happiness, and satisfaction in the clinical study

In the clinical study, the participants recorded changes in the VAS scores for their stress, happiness, and satisfaction. This paper suggests using this method for SM classification. Stress, happiness, and satisfaction are independent variables in humans [38], and stress relief (i.e., relaxation), happiness, and satisfaction are very adjacent to each other in the emotional space, which is represented by the arousal-valence relationship [39]. Therefore, if the CNN model can classify SM correctly, stress relief’s effect is more significant than happiness and satisfaction, and the degree of difference would be clear. In Section 4.2, Fig 6 demonstrates that the VAS scores for stress doubly decreased compared to the VAS scores for happiness and satisfaction.

### 5.4 Limitations and future directions of this study

While promising, this study encounters several limitations that pave the way for future research directions.

Firstly, the focus on Korean participants and music limits the generalizability of our findings. To establish the broader applicability of our CNN model with MSSs in classifying SM, future research should aim to include a diverse range of cultural contexts and musical genres. This expansion will help in understanding the cross-cultural effectiveness of our method and the customized characteristics of SM.

Secondly, our analysis addressed the short-term impacts of CNN-classified SM. While we observed positive immediate effects such as stress reduction and enhanced satisfaction, the long-term impacts of regular SM consumption remain unexplored. Future studies should delve into these long-term effects to understand how sustained exposure to CNN-classified SM influences emotional well-being and stress levels over time [40].

In addition to these limitations, ethical considerations play a crucial role, particularly when personalizing stress-relief music based on individual preferences. The personalization process involves handling sensitive individual data, necessitating strict adherence to privacy, consent, and data security principles. Informed consent must be obtained from participants, ensuring that personal data is anonymized and protected. Moreover, the potential psychological impacts of personalized music therapy should be carefully evaluated to safeguard participants’ mental health.

Despite these limitations, this study contributes significantly to the field of music therapy. The development of a simplified approach to classify SM using CNNs and MSSs opens avenues for applying this methodology in varied cultural settings. Additionally, our initial findings on the short-term effectiveness of SM provide a foundation for more comprehensive studies. These future investigations should focus on both the short-term and long-term effects of personalized SM on diverse populations, enhancing our understanding of the therapeutic potential of music in emotional well-being and stress management.

## 6 Conclusion

This paper introduced a novel deep learning approach using convolutional neural networks (CNNs) to construct datasets of stress-relief music (SM), overcoming the limitations of traditional methods that rely on measuring biological responses. Unlike previous studies that were constrained by time-consuming, costly, and equipment-dependent processes, our method utilizes elements of sound—frequency, amplitude, and waveform—directly extracted from music. These elements were transformed into Mel-scaled spectrograms, leveraging the proven efficacy of CNNs in music genre classification to enhance time efficiency and reduce costs.

A key contribution of this study is the demonstration of the CNN model’s remarkable ability to identify SM with a 98.7% test accuracy, showcasing its potential across various musical genres. Additionally, the clinical study validated the effectiveness of the machine learning-selected music, establishing its comparability with researcher-verified music in terms of satisfaction, happiness, and stress relief. This outcome not only confirms the practical utility of our approach but also underscores its potential applicability beyond the scope of conventional methods.

While the technical aspects of using CNNs for music classification may align with existing methodologies, the application of these techniques in the context of SM selection represents a significant advancement. By validating our approach through a clinical study, we bridge a significant gap in music therapy research, offering a scalable, efficient, and cost-effective method for creating diverse and personalized SM datasets. This approach holds promise for enhancing the effectiveness of music therapy and could be applied to other domains within music and sound therapy. Future research can build upon these findings to explore the broader implications of music in emotional well-being and stress management, potentially transforming practices in music therapy and patient care.

## Supporting information

S1 Appendix (PDF)
