# Supplementary material for: Accelerated construction of stress relief music datasets using CNN and the Mel-scaled spectrogram
Source: PLoS One. 2024 May 24;19(5):e0300607. doi: 10.1371/journal.pone.0300607 (PMC11125514; doi:10.1371/journal.pone.0300607)
Supplement: S1 Appendix — (PDF) [file pone.0300607.s001.pdf]

A Dataset Description for Clinical Study

Table 8. List of top Korean songs and their singers used in the clinical study. (Part 1)

Index	Song	Artist
1	How can I love the heartbreak, you're the one I love	AKMU
2	In full bloom	Kim Ho-joong
3	I Should Probably Go To Bed	Dan + Shay
4	Painkiller	Judas Priest
5	Mood	24kGoldn
6	Your Eyes Tell	BTS
7	Honesty	Billy Joel
8	Start Over	Gaho
9	Stay Gold	BTS
10	Flowers	Markers
11	Beach Again	Lee Hyori
12	Speechless	Naomi Scott
13	ROCKSTAR	Post Malone
14	Someone Like You	Adele
15	Everglow	Coldplay
16	BLOOM	M.C the MAX
17	Zenzenzense	Radwimps
18	CrocodileSong	Pinkfong
19	You Belong to Me	Carly Simon
20	Head, Shoulder, Knees	Pinkfong
21	The Moment My Heart	Kyuhyun
22	All Falls Down feat. Juliander	Alan Walker
23	Slightly Tipsy	Sandeul
24	right here	SWV
25	Keep Being You	Isyana Sarasvati
26	See You Again	Wiz Khalifa
27	God, He Shares Our Pain	WELOVE
28	Into the Unknown	AURORA and Idina Menzel
29	EX	Kiana Ledé
30	Sweet on You	Teresa Teng
31	Flower Dance	DJ Okawari
32	Angels We Have Heard on High	Andrea Bocelli
33	Love, Seasons	Song So-hee
34	Home	Edward Sharpe and the Magnetic Zeros
35	Paris In The Rain	Lauv
36	Thinking Out Loud	Ed Sheeran
37	Love Affair	UMI
38	The Blower's Daughter	Damien Rice
39	Way Maker	Markers
40	Lost Stars	Adam Levine
41	Despacito	Luis Fonsi
42	Reminiscent	Yiruma
43	WAP	Cardi B
44	Late Night	Noel
45	Oh Happy Day	Edwin Hawkin Singers
46	bad guy	Billie Eilish
47	Spring day goes	Kim Yuna
48	The Ocean	Mike Perry
49	Viva La Vida	Coldplay
50	BANANA CHACHA	Momoland
51	Autumn Leaves	Joseph Kosma
52	Goodbyes	Post Malone
53	Believer	Imagine Dragons
54	Purple color post card	Lim Young-woong
55	Nandemonaiya	Radwimps

Table 9. List of top Korean songs and their singers used in the clinical study. (Part 2)

Index	Song	Artist
56	Dance Monkey	Tones and I
57	The Time, Penetrated	WELOVE
58	West Coast Love	Emotional Oranges
59	Stalker	10cm
60	Payphone	Maroon 5
61	My Grandfather's Clock	Johnny Cash
62	We are all Muse	Seok Chul Yun
63	Show Yourself	Evan Rachel Wood and Idina Menzel
64	Alive	Big Z
65	Heart	Taeyeon
66	Comfortable	Steve Void & TELYKast
67	Desce Pro Play	Anitta, MC Zaac, and Tyga
68	Every day, Every Moment	Paul Kim
69	LOVE me	BE'O
70	Don't Know Why	Norah Jones
71	Don't Start Now	Dua Lipa
72	Maniac	Conan Gray
73	24H	SEVENTEEN
74	Arirang alone	Kim Ran Young
75	Lemon	Kenshi Yonezu
76	Introduce me a good person	Joy
77	Sugar	Maroon 5
78	Into the Unknown	TAEYEON
79	Youngblood	5 Seconds of Summer
80	For A Minute	WizTheMc
81	Tight	10cm
82	Something Just Like This	Coldplay and The Chainsmokers
83	Together	SEVENTEEN
84	At the Place Where You Call	Markers
85	Bom	Bolbbalgan4
86	Take care	MRB
87	Be Kind	Halsey and Marshmello
88	Whiskey and Morphine	Alexander Jean
89	A Little Happiness	Hebe Tien
90	CAT	Sunwoo Jung A
91	Circles	Post Malone
92	Quando, Quando, Quando	Engelbert Humperdinck
93	I Knew I Love	Jeon Mi-do
94	Love song	Seok Chul Yun
95	Sleeping Beauty	Paul
96	Despacito	Lewis Figi
97	Stuck with U	Ariana Grande and Justin Bieber
98	Kiss The Rain	Yiruma
99	Let Her Go	Passenger
100	No umbrella	Kim Ho-joong
101	May Be	Yiruma
102	Love is	Jeon Sang Keun
103	Closer	The Chainsmokers
104	Blinding Lights	The Weeknd
105	A Wild Rose	Baek Nan-ah
106	Blind Love	Ensemble Sinawi
107	I still love you a lot	Baek Ji-young
108	Why are you come out there	Young Tak
109	Beautiful Korea	Song So-hee
110	Happiness	Red Velvet

Table 10. List of top Korean songs and their singers used in the clinical study. (Part 3)

Index	Song	Artist
111	Phonecert	10cm
112	Rainbow	Kacey Musgraves
113	Dynamite	BTS
114	Crush	Moon Seong-wook
115	All for One	Sookmyung Gayageum Orchestra
116	Jochebed'ssong	Yum Pyung-Ahn
117	Aloha	Jo Jung-suk
118	ROXANNE	Arizona Zervas
119	Hello	Joy
120	Just as I Am	J-US
121	Drink Makgeolli	Young Tak
122	Yue Liang Dai Biao Wo De Xin	Teresa Teng
123	Maria	Hwasa
124	I Love You 3000	Stephanie Poetri
125	Last Night	Joseon blues
126	Lullaby	Brahms
127	From Ish, To Isha Chords	Kim Bokyu
128	You've Got a Friend in Me	Randy Newman
129	Faded	Alan Walker
130	River Flows In You	Yiruma
131	The song of separation	2nd Moon
132	On My Way	Alan Walker, Farruko, and Sabrina Carpenter
133	Someday, The Boy	Kim Feel
134	2 soon	Keshi
135	Dear my blue	Baek Yerin
136	blue	Eiffel 65
137	2002	Anne-Marie
138	Say So	Doja Cat
139	Baby Shark	Pinkfong
140	Old Song	Standing Egg
141	12:45 (Stripped)	Etham
142	247	SEVENTEEN
143	The tomato song	Pinkfong
144	Day 1 ◑	HONNE
145	Ssukdaemeori	Park Ae-ri
146	My Desire and Prayer	Min Ho ki
147	Give You My Heart	IU
148	Autumn morning	IU
149	The Bones	Maren Morris
150	Confession Is Not Flashy	Kyuhyun
151	Bad Boy	Yung Bae
152	Pinwheel	SEVENTEEN
153	death bed	Powfu
154	10,000 Hours	Dan + Shay and Justin Bieber
155	Three Bears	Pinkfong
156	22	Taylor Swift
157	ily (i love you baby)	Surf Mesa
158	Thunder	Imagine Dragons
159	Leo	Bolbbalgan4
160	To My Youth	Bolbbalgan4
161	Lullaby	Iksoo Shin
162	Your Shampoo Scent In The Flowers	Jang Beom-june
163	Memories	Maroon 5
164	Sunflower	Post Malone and Swae Lee
